# Supplementary material for: Space-charge-limited electron and hole currents in hybrid organic-inorganic perovskites
Source: Nat Commun. 2020 Aug 11;11:4023. doi: 10.1038/s41467-020-17868-0 (PMC7419305; doi:10.1038/s41467-020-17868-0)
Supplement: Supplementary file 1 — Supplementary Information [file 41467_2020_17868_MOESM1_ESM.pdf]

Supplementary Information

Space-charge-limited electron and hole currents in hybrid organic-inorganic perovskites

Mohammad Sajedi Alvar, Paul W. M. Blom, and Gert-Jan A. H. Wetzelaer

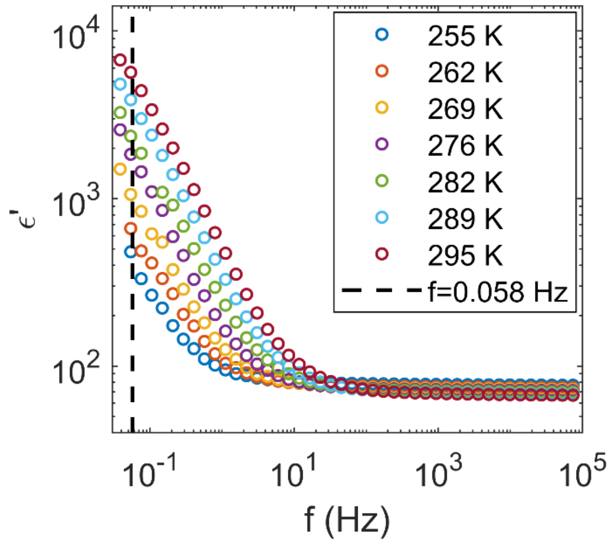

Fig. S1 | Permittivity of $MAPbI_3$ thin film at different temperatures obtained by impedance spectroscopy. The low frequency permittivity significantly decreases by decreasing the temperature, which is accordance with the temperature dependence of ion diffusivity. High frequency exhibits a slight increase upon decreasing the temperature.

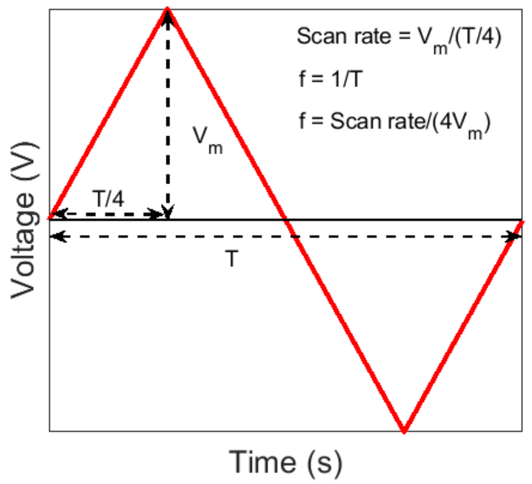

Fig. S2 | Applied voltage for current voltage measurements. The correlation between the scan rate and frequency is presented.

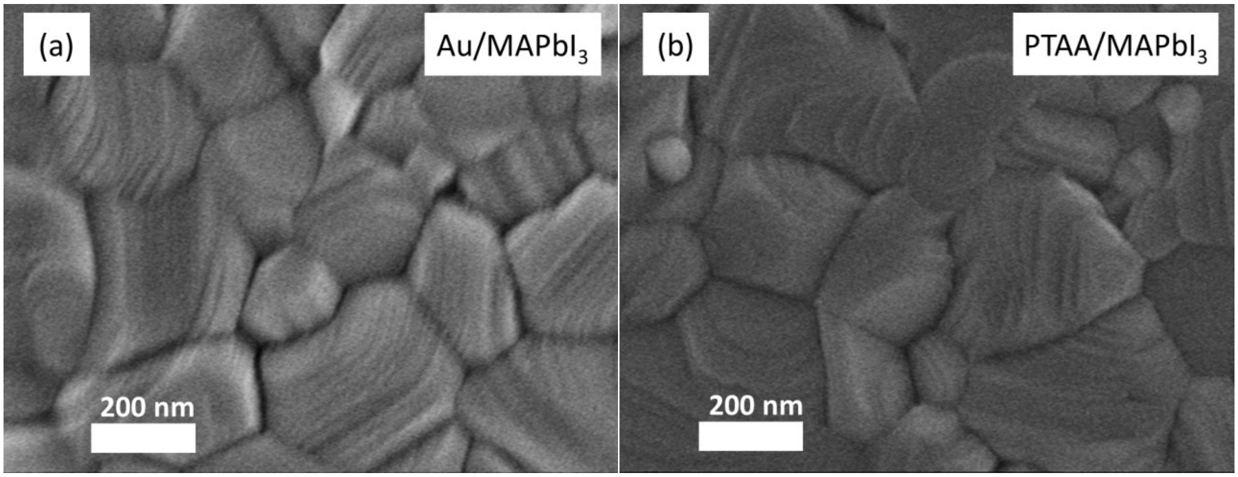

Fig. S3 | Scanning electron microscopy images of the perovskite film on top of Au (a) and PTAA (b).

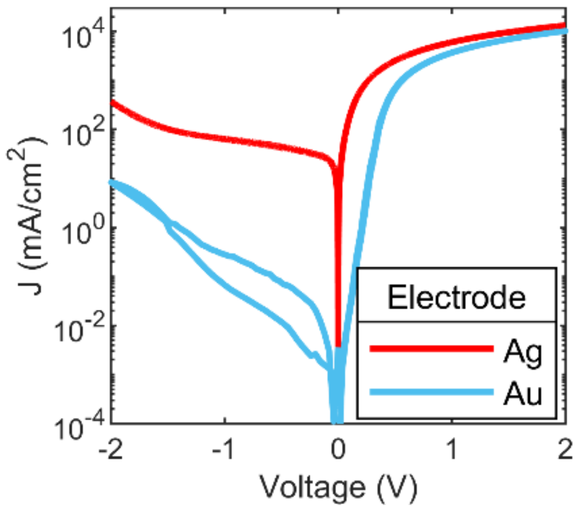

Fig. S4 | Current density-voltage characteristics of electron-only devices with different bottom electrodes. The work functions of Ag (4.6 eV) and Au (5.0 eV) result in different electron-injection barriers in reverse bias, which give rise to differences in the reverse-bias current and the built-in voltage. This shows that the asymmetric current-voltage characteristics in the electron-only devices are caused by the barrier at the bottom electrode.

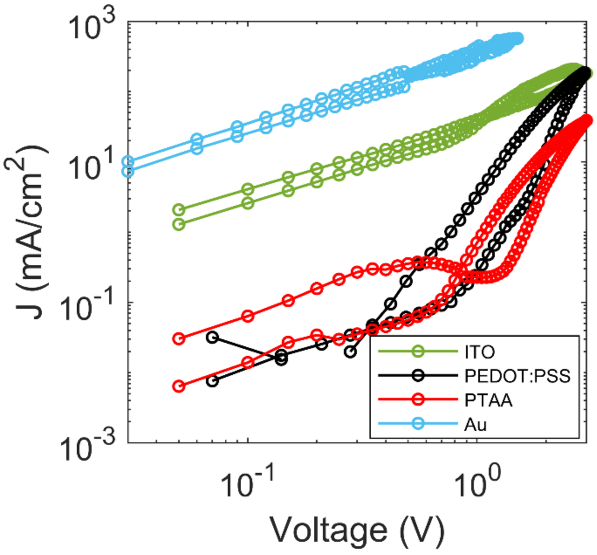

Fig. S5 | Comparison of hole injection from different hole injection layers. The Au contact shows the highest current as compared to ITO, PEDOT:PSS and PTAA. Furthermore, at low voltage the current density for the Au contact shows a linear dependence on voltage, whereas the injection-limited currents from the other electrodes show a steeper dependence.

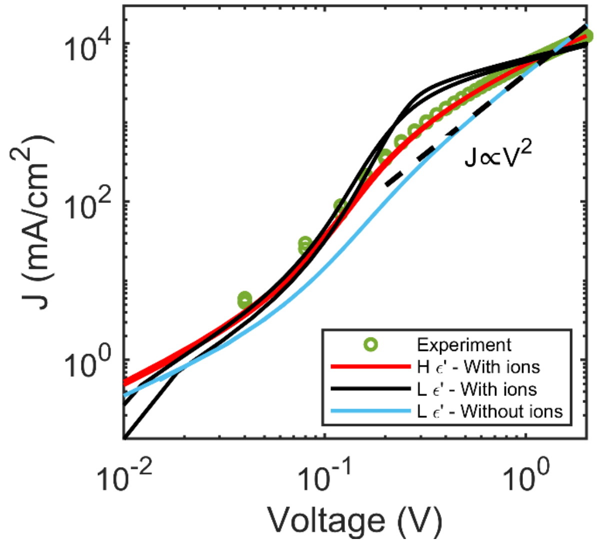

Fig. S6 | Experimental (symbols) and modelled electron-only current. The modelled electron currents include a high permittivity and ion movement (red line), a low permittivity and ion movement (black line) and a low permittivity without ions (classical SCLC model) (blue line).

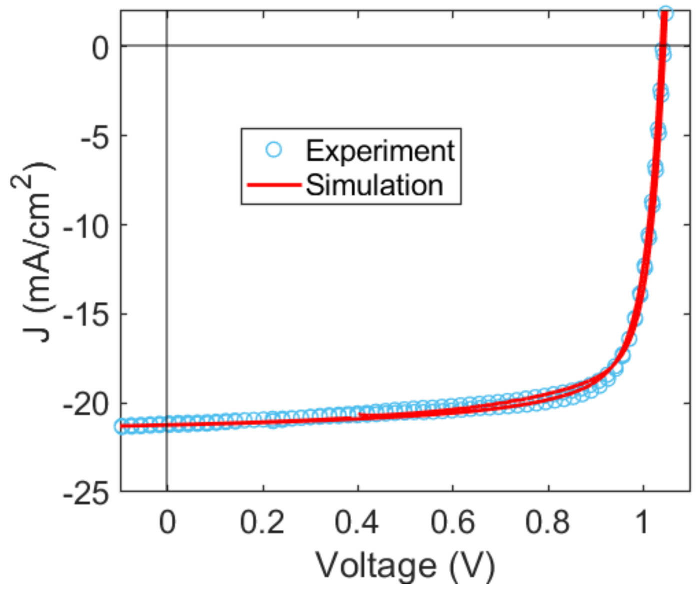

Fig. S7 | Current density-voltage characteristics of an ITO/PTAA/$MAPbI_3$/C_{60}/TPBi/Al solar cell. The experimental characteristic (symbols) shows a decent open-circuit voltage, short-circuit current density, and fill factor. The simulation reproduces the solar cell characteristics using the obtained mobilities, assuming Langevin-type recombination with a Langevin reduction factor of 0.01. This preliminary simulation result demonstrates that the solar cell can be simulated with the obtained mobilities and a low recombination coefficient. A more detailed analysis of the recombination mechanisms and solar-cell device physics will be the subject of future studies.

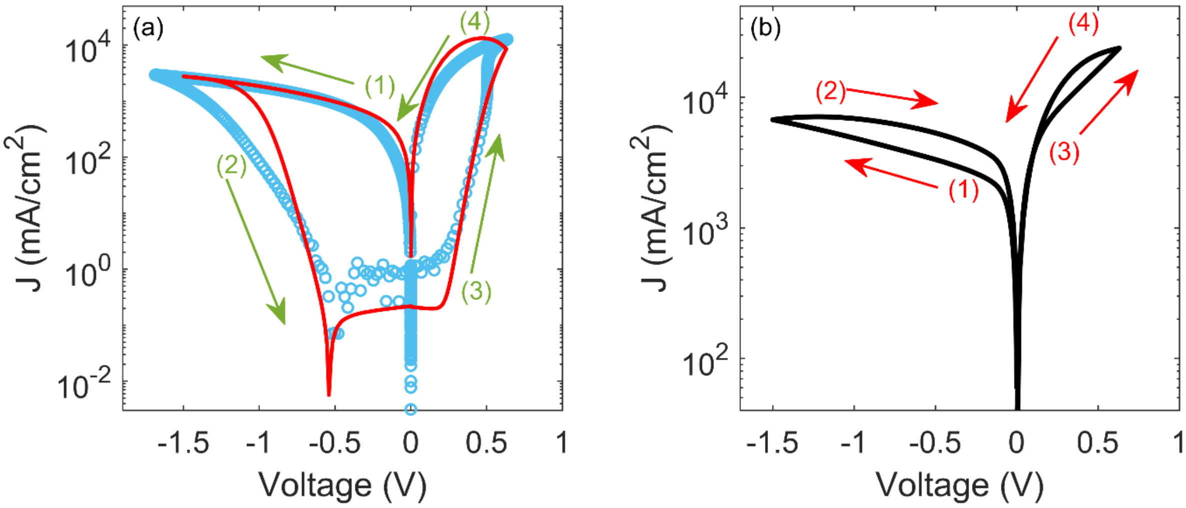

Fig. S8 | Experimental (symbols) and calculated current density-voltage characteristics of an electron-only device at 295 K at a high scan rate (100 V/s). The electron current is simulated with the same set of parameters under two different conditions for ionic charges: mobile positive ions and a uniform distribution of immobile negative ions (a) and mobile negative ions and a uniform distribution of immobile positive ions (b).

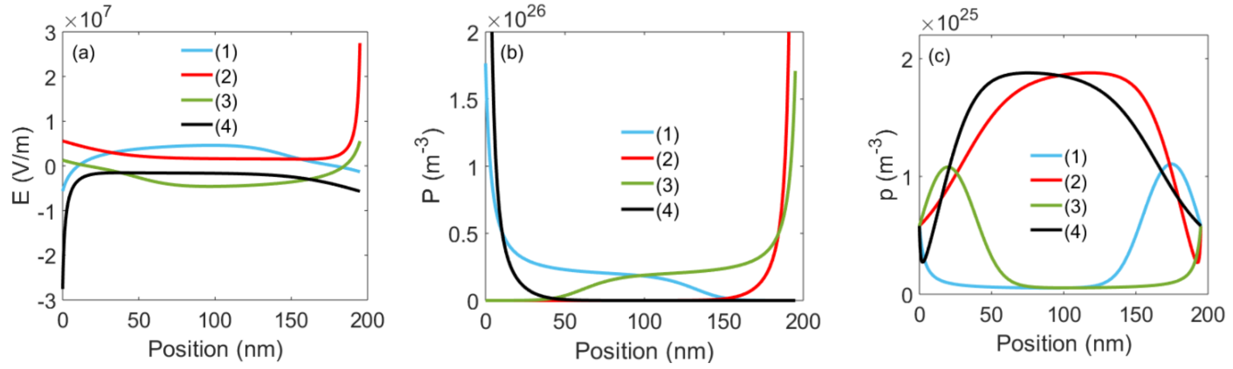

Fig. S9 | Electric field, hole and ion concentration profiles. The electric fields (a), hole (b) and ion (c) distributions for mobile positive ions at forward (blue and red) and reverse (green and black) bias of 0.5 V corresponding to the Fig. 5a.

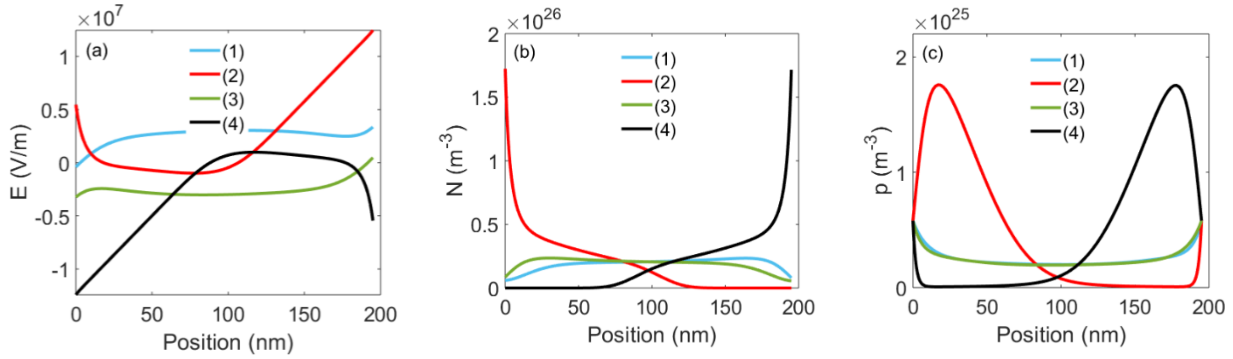

Fig. S10 | Electric field, hole and ion concentration profiles. The electric fields (a), hole (b) and ion (c) distributions for mobile negative ions at forward (blue and red) and reverse (green and black) bias of 0.5 V corresponding to the Fig. 5b.
